# A Novel Pan-*Flavivirus* Detection and Identification Assay Based on RT-qPCR and Microarray

**DOI:** 10.1155/2017/4248756

**Published:** 2017-05-24

**Authors:** Ariel Vina-Rodriguez, Konrad Sachse, Ute Ziegler, Serafeim C. Chaintoutis, Markus Keller, Martin H. Groschup, Martin Eiden

**Affiliations:** ^1^Institute for Novel and Emerging Infectious Diseases, Friedrich-Loeffler-Institut (Federal Research Institute for Animal Health), Insel Riems, Greifswald, Germany; ^2^Institute of Molecular Pathogenesis, Friedrich-Loeffler-Institut (Federal Research Institute for Animal Health), Jena, Germany; ^3^Diagnostic Laboratory, Department of Clinical Sciences, School of Veterinary Medicine, Faculty of Health Sciences, Aristotle University of Thessaloniki, Thessaloniki, Greece

## Abstract

The genus* Flavivirus* includes arthropod-borne viruses responsible for a large number of infections in humans and economically important animals. While RT-PCR protocols for specific detection of most* Flavivirus *species are available, there has been also a demand for a broad-range* Flavivirus* assay covering all members of the genus. It is particularly challenging to balance specificity at genus level with equal sensitivity towards each target species. In the present study, a novel assay combining a SYBR Green-based RT-qPCR with a low-density DNA microarray has been developed. Validation experiments confirmed that the RT-qPCR exhibited roughly equal sensitivity of detection and quantification for all flaviviruses tested. These PCR products are subjected to hybridization on a microarray carrying 84 different oligonucleotide probes that represent all known* Flavivirus* species. This assay has been used as a screening and confirmation tool for* Flavivirus* presence in laboratory and field samples, and it performed successfully in international External Quality Assessment of NAT studies. Twenty-six* Flavivirus* strains were tested with the assay, showing equivalent or superior characteristics compared with the original or even with species-specific RT-PCRs. As an example, test results on West Nile virus detection in a panel of 340 mosquito pool samples from Greece are presented.

## 1. Introduction

The genus* Flavivirus* contains nearly 70 recognized viruses, many of which infect humans and economically important animals [1]. Flaviviruses, such as Dengue virus (DENV) [2] and Yellow fever virus (YFV) [3], have been a common cause of devastating diseases in tropical and less developed countries, but in recent years the emergence of flaviviral zoonoses was observed worldwide. Examples include the occurrence of West Nile virus (WNV) in the United States [4], Japanese encephalitis virus (JEV) in Australia [5], and Usutu virus (USUV) [6], WNV [7], and DENV [2] in Europe. Recently, Zika virus (ZIKV) also expanded into Southern America, with reports of detection in Europe and USA [8].

Large surveillance and early warning systems commonly applied in European countries and around the world could benefit from a more sensitive and broader range screening method. Both mosquito pools and (sentinel) birds are common targets of massive screening for arbovirus, particularly for flaviviruses like WNV or USUV [6, 9, 10]. Rapid virus identification and quantification are crucial for accurate diagnosis of ongoing infections, treatment selection, and follow-up, as well as for selection and timely introduction of control measures in outbreaks scenarios. In this context, highly parallel detection technologies, such as DNA microarrays, are gaining importance [11–20].

Like RNA viruses in general, flaviviruses are distinguished by extensive genetic heterogeneity, which implies classification into subunits, for example, genotypes and lineages, each with distinct epidemiological or clinical significance. This heterogeneity represents a major challenge in primer and probe design for PCR and DNA microarray assay development.

In the present study, we have optimized a pan-*Flavivirus*-specific SYBR Green-based real-time reverse-transcription polymerase chain reaction (RT-qPCR) [21]. The amplification is subsequently complemented with hybridization on a low-density DNA microarray, which exploits the genetic heterogeneity contained in the internal segment of the PCR amplicon, thus allowing rapid identification of flaviviruses from clinical or field samples.

## 2. Materials and Methods

### 2.1. Viral Samples and RNA Extraction

The 26* Flavivirus* strains to be used as reference were collected and stored at −80°C until being processed as follows.

African green monkey kidney (Vero) cells (Collection of Cell Lines in Veterinary Medicine, Friedrich-Loeffler-Institut, Insel Riems, Greifswald, Germany) were infected with viruses in biosafety L3^*∗∗*^ laboratory facilities, and cell culture supernatants were collected and inactivated in Buffer AVL (QIAGEN, Hilden, Germany) as previously described [22, 23]. The WNV strains lineage 1a-NY99 (ac. AF196835) and Dakar, lineage 1b-Kunjin (ac. D00246) and lineage 2-Uganda 1937 (ac. M12294), B956 (ac. AY532665), Sarafend (ac. AY688948), and goshawk Austria 361/10 (2009) (ac. HM015884) were reused from our previous works [22–24]. The strains USUV Germany 2011 (BH/65) (ac. HE599647), YFV 17D, JEV, TBEV Langat, Malaysia, 1956 (ac. AF253419), and Murray Valley encephalitis virus (MVEV) were reused from another work [25]. USUV Austria 2001 (939/01) and Germany 2011 (939/01) were taken from [26] and JEV Nakayama (ac. EF571853) and TBEV Neudoerfl (ac. U27495) from [27].

Additionally, the following strains were obtained from the Health Protection Agency, Salisbury, United Kingdom: WNV lineage 2, MB 1957; SLEV; YFV “French Neurotrop” and LolIl (#780). JEV Nakamura was kindly provided by A. Müllbacher (John Curtin School of Medical Research, Canberra, Australia). RNA from ZIKV and DENV 1, 2, 3, and 4 viruses was provided by P. Despres (*Flavivirus* Unit, Institute Pasteur, Paris, France), while F. Hufert and M. Weidmann (Institute for Virology, Göttingen, Germany) kindly provided the viruses TBEV Absettarov, 1951 (ac. AF091005), TBEV Aina, 1963 (ac. AF091006), and TBEV Hypr, 1953 (ac. U39292).

RNA was extracted with the RNeasy Mini Kit, or the QIAamp Viral RNA Mini Kit (QIAGEN, Hilden, Germany), according to the manufacturer's protocol. A synthetic RNA was used as internal extraction control (IC) [28]. The NY99 RNA was used to create an external calibrator curve, which in turn was calibrated using the 5′UTR WNV-specific RT-qPCR [22].

### 2.2. Selection of Primers and Probes

Nearly 200 complete* Flavivirus* genome sequences were obtained from the NCBI Nucleotide database by the end of 2010. Very similar sequences (more than 98% identity) were excluded. The sequences were aligned using CLUSTAL X [29] (accessed from BioEdit software v.7.0.5.3) [30] and VectorNTI Advanced v.10 (Invitrogen, Carlsbad, CA, USA). This alignment was manually curated using both the nucleotide and the deduced amino acid sequences and extended by adding partial sequences to reach a total of more than 400, so as to represent the NS5 gene of all known species, including most subtypes or lineages (a regularly updated version is available at* Flavivirus GitHub site *(https://github.com/qPCR4vir/Flavivirus)).

*VisualOligoDeg* (https://github.com/qPCR4vir/VisualOliDeg) was used to facilitate visualization and selection of appropriate oligonucleotides hybridizing to sequences of a given. The NS5 regions (spanning nucleotides 9040 to 9305 of AF196835) of the aligned sequences were imported into this workbook and were manually classified into major groups (MB: mosquito borne; TB: tick borne; and insect-only), virus groups (JEVG, YFVG, TBEVG, etc.), species (WNV, JEV, YFV, TBEV, etc.), and in some cases lineages (like WNV-1 or WNV-2, etc.) or genotypes.

Alongside species-specific RT-PCRs [31], broader, genus-specific RT-PCR protocols have been also reported [19, 21, 32, 33]. A genus-specific Taq-Man RT-PCR for* Flavivirus* detection was modified [21], with primer sequences optimized using* VisualOligoDeg.* It targets conserved flanking regions with an internal region with sufficient variability to enable virus identification by sequencing the amplicon. The original sequences were adapted to consistently amplify most of the* Flavivirus* members resulting in degenerate primers PFlav-fAAR (TACAACATGATGGGAAAG**A**GAGAGAA**R**AA from 9040 to 9068 of AF196835) and PFlav-rKR (GTGTCCCA**K**CC**R**GC**T**GTGTCATC from positions 9305 to 9283 of AF196835).

A total of 50 probes with a Tm around 55°C were selected. A second set of sequences was also selected as a replacement, in case of failure of first-set sequences. The candidate sequences were submitted to the manufacturer for a final in silico evaluation of properties (homogenous hybridization, discriminatory potential, etc.). As a result, all 84 sequences were found suitable for inclusion in the production of the microarray (Table S01 in Supplementary Material available online at https://doi.org/10.1155/2017/4248756).

### 2.3. Detection and Quantification Using RT-qPCR

A one-step SYBR Green-based RT-qPCR with melting curve analysis was developed. The QuantiTect SYBR® Green RT-PCR Kit (QIAGEN, Hilden, Germany) was used following the manufacturer's instructions. Briefly, each primer (PFlav-fAAR and PFlav-rKR) was 5′-biotinylated during the initial synthesis (Eurofins Genomics, Ebersberg, Germany) and used at a final concentration of 0.8 *μ*M, in a final reaction volume of 25 *μ*L, including 5 *μ*L of RNA sample solution. Real-time RT-qPCR was carried out on a CFX96 real-time PCR detection system (Bio-Rad Laboratories, Hercules, USA). The thermal cycling profiles are presented in Table 1. Species-specific RT-qPCR were also used to determine the sensitivity of the* Flavivirus* RT-qPCR. Reference RNA samples included WNV [22], USUV [26, 34], and TBEV [35]. In order to have comparable Cq values across experiments, when possible, we set a separate fluorescence cut-off value for each target (PCR primer mix) in each 96-well plate run, such that Cq ≈ 28 for the control RNA WNV NY99 diluted 10^−4^. This control was previously calibrated using a synthetic RNA [22]. In order to perform* Flavivirus* quantification, an external standard curve consisting of four dilutions of RNA WNV NY99, 10^−2^, 10^−3^, 10^−4^, and 10^−5^, was included in each run. These were equivalent to 4 × 10^4^, 4 × 10^3^, 4 × 10^2^, and 40 genome copies/*μ*L of RNA solution or to 1.4 × 10^7^, 1.4 × 10^6^, 1.4 × 10^5^, and 1.4 × 10^4^ copies per mL of homogenized sample, respectively. To analyze the* Flavivirus* SYBR Green RT-PCR, the fluorescence was measured at step (6) of the standard protocol (Table 1). Similarly, for species-specific RT-qPCR, fluorescence measurement was conducted at step (4).

### 2.4. Sequence Analysis

Twenty *μ*L of nonpurified PCR product was sent to Eurofins Genomics (Ebersberg, Germany) for direct DNA sequencing by the Sanger dideoxy method, using the amplification primers. A BLAST (NCBI) search of the obtained sequences was usually sufficient to identify the virus strain. The (internal) amplicon of approximately 240 nt contains sufficient phylogenetic information to reconstruct phylogenetic trees and allows classification of more distantly related and unknown strains and even new species.

### 2.5. Microarray Analysis

*Flavivirus*-specific oligonucleotide probes selected using the* VisualOligoDeg* were spotted onto a low-density microarray of the commercially available ArrayStrip™ (AS) platform (Alere Technologies GmbH, Jena, Germany). The 84 probes (spots 1–84 in S01 File) were spotted either in triplicate (Chip Wildtech Virology-Mycob 01, from 2011-01-13, assay ID-10610) [36] or in quintuplicate (Chip Wildtech Virology 02, from 2012-09-10, assay ID-16050). The Alere Hybridization Kit was used following previously published instructions [37, 38]. Briefly, only positive RT-PCR reactions were routinely analyzed, from which 1 *μ*L was directly denatured in 100 *μ*L of the hybridization buffer at 95°C for 5 min and then placed for cooling at 4°C for 5 min in a thermocycler (Bio-Rad Laboratories). This solution was transferred to the AS vessel (previously conditioned with water and hybridization buffer) and incubated at 55°C for 1 h upon shaking at 550 rpm on a BioShake iQ heatable shaker (Quantifoil Instruments, Jena, Germany). The AS vessel was subsequently washed twice at 50°C for 10 min, incubated with 100 *μ*L of a peroxidase conjugate solution at 30°C for 10 min, and washed and incubated at room temperature with 100 *μ*L of the substrate solution (Seramun Grün; Seramun Diagnostica GmbH, Heidesee, Germany) for 5 min. Images of processed microarrays were saved in bitmap (.bmp) format, using the ArrayMate transmission Reader (Alere Technologies GmbH).

### 2.6. Microarray Data Processing and* Flavivirus* Identification

The web-based database* Pionir, The Experiment Navigator of Partisan Array LIMS* (Alere Technologies GmbH), was used for visualization and analysis of images and complete experiments, as well as for additional backup. Alongside, the Partisan IconoClust® v3.6r0 software (Alere Technologies GmbH) was used for local analysis of microarray images, generating for each spot of each picture the background-corrected signal intensities NI = 1 − *M*/BG, with NI being normalized intensity, *M* average (mean) spot intensity, and BG local background intensity. Spot intensities are measured as light transmission, with *M* values ranging from 1 for complete transmission (background, weak spots) to 0 for complete absorption (dark spots). Thus, normalized signal intensities range between 0 and 1. Previous evaluation of the signal to background ratio (SBR) in assays using the same technology has set the cut-off value for positive signals to 0.1 [37]. A* custom python script* (https://github.com/qPCR4vir/Flavivirus/blob/master/microarray/icono_clust_scripts/OnlyMeanSignal.icrun) was embedded in this software to enable visualization of individual hybridizations or batch analysis of series of experiments. This script exports experimental data in different formats, including a format suitable for import into* Orange *software, which allows visual programming and python scripting for data mining and visualization (v2.7.6.dev, installed 2014-06-06 from http://orange.biolab.si/).

Using* Orange*, the visual program* PanFlavExpStdSampl* (https://github.com/qPCR4vir/Flavivirus/tree/master/microarray/orange) (Figure S01) was created, which, together with custom modifications of some* parts of Orange *(https://github.com/qPCR4vir/orange/commit/84f0a20e58b40b238f52319f2017ae77df0dbf72) itself, permits an interactive import of the experiments used as standards (known samples) for parallel analysis with unknown samples for classification. This program calculates the distances between pairs of signal intensity patterns (two microarray experiments), which can serve as a measure of similarity between samples. Several distance formulas are available interactively from* Orange* and their meaning is explained elsewhere [39]. These distances are visualized in one of the widgets as heat-map-like graphics (Figure 5), in which the labels and the order of the samples can be interactively selected from a group of preoptions. We have added (directly* modifying the source code of Orange* (https://github.com/qPCR4vir/orange/commit/21c3996c1712baf8819e2050e7dccc31593cf2a0)) the option to reorganize the heat map showing the selected sample at the top followed by the most similar samples by mouse-clicking the respective cell. This graphic can also show the samples organized in a tree to reveal clustering. Another* Orange *widget uses the distances to construct a tree (Figure 4), in which a cut-off can be interactively selected, and/or groups defined to make a report of the proposed classification.

### 2.7. Field Samples

After development, initial evaluation, and optimization, the new assay was further evaluated by testing RNA extracts from mosquito pools from Greece. Mosquito trapping was performed within the framework of a surveillance program, which was implemented in the region of Central Macedonia during the 2012 arbovirus transmission period. The aims of this program were to molecularly characterize WNV and assess population dynamics of the major arbovirus vector species, to timely notify public health authorities on increased risk [40]. Female adult mosquitoes were collected at dry ice-baited CDC mosquito traps, which were set in areas with previous indications of high arbovirus prevalence [40].

Identified mosquitoes (50 individuals of the same genus) were placed in 2 mL microcentrifuge tubes with two 4 mm sterile glass balls and 1 mL of phosphate-buffered saline (PBS) containing 1% of heat-inactivated fetal bovine serum (FBS; Sigma-Aldrich, Steinheim, Germany). Disruption was performed for 30 s at speed 4.0 using a RiboLyser homogenizer (Hybaid, Ltd., Teddington, UK). The homogenates were centrifuged (16,000 ×g, 5 min at 4°C), and 150 *μ*L of supernatant from each mosquito pool underwent RNA isolation, based on the previously described RNA extraction method.

In total, 340 mosquito pools were tested with the* Flavivirus *RT-qPCR protocol. Out of them, 180 pools were represented by* Culex* mosquitoes, 75 were comprised by* Aedes,* and 85 were comprised by* Anopheles* mosquitoes.

## 3. Results

**Figure 1:**
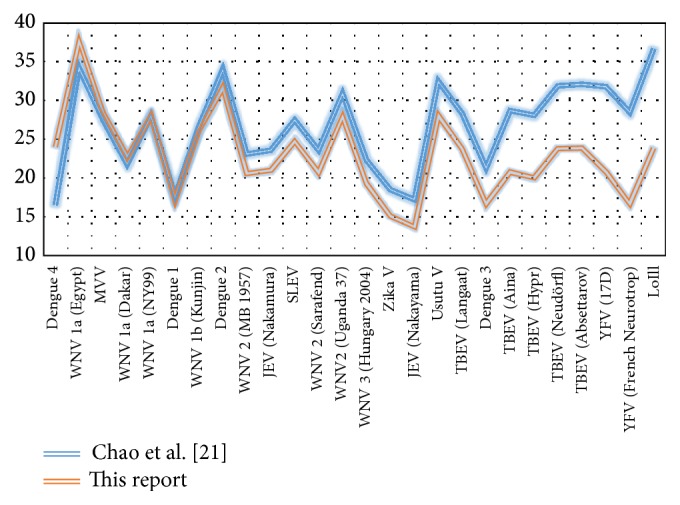
Cq values for 26 viruses. Comparison of Cq values obtained for different viruses using the protocol of Chao et al. [21] (blue line) with those of our own procedure (red line). The same virus RNA preparation was tested in the same PCR plate with both RT-qPCR protocols. The order of viruses from left to right is according to increasing Cq difference.

Following preliminary observations that the sensitivity and efficiency of amplification with the primers from [21] were not homogeneous for different flaviviruses, we decided to design a modified set of degenerate primers. Using* VisualOligoDeg* we selected primers for a modified RT-qPCR for* Flavivirus *detection and quantification. In the initial experiments, we compared the new procedure with published RT-qPCRs using reference viruses. All 26* Flavivirus* reference strains described in Section 2.1 were tested (Figure 1).

The optimized* Flavivirus* RT-qPCR was also compared in terms of detection limit with species-specific RT-qPCRs for WNV, USUV, and TBEV by testing fresh RNA solutions (from supernatants of infected cells) in a series of end-point dilution experiments (Figures S02 and S03).

**Figure 2:**
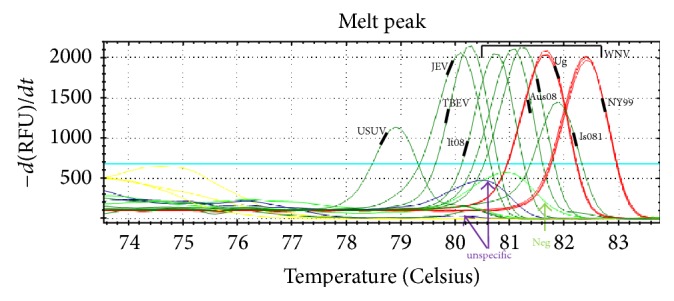
Melting curves from different flaviviruses. Melting curve analysis of the amplicons from different flaviviruses (samples from the ANSES WNV Proficiency Test, 2013). NY99, It08, and Is985 are WNV lineage 1a strains, while Aus08 is a lineage 2 strain. The viruses were identified using the microarray, with confirmation by DNA sequencing.

Subsequently, we conducted melting curve analysis, which can provide useful information to determine positivity and sequence differences (as shown in Figure 2) but which has limited value for identification. A well-defined peak between 79 and 84°C is a strong indication of positivity, and differences in Tm indicate sequence differences, possibly representing different species or lineages. The results from end-point dilution experiments and the calculations using the externally calibrated standard curve suggest that, for most members of* Flavivirus *genus, this RT-qPCR assay is highly sensitive, capable of detecting a few viral RNA copies per reaction. However, this also means that cross-sample contamination and DNA carryover are a major concern. Therefore, it is crucial to organize the laboratory work accordingly and include sufficient controls to validate the results of each experiment.

To evaluate the accuracy and performance of the developed RT-qPCR assay, we successfully participated in five international ring trials for quality assessment of nucleic acid amplification tests, that is, ANSES 2013 (melting curves of the samples shown in Figure 2), Quality Control for Molecular Diagnostics or QCMD-2010QCMD-2011 [41], QCMD-2012, and QCMD-2013 (results summarized in Table S02) (http://www.qcmd.org/). These results complemented the information obtained from WNV-specific RT-qPCRs. Both WNV and non-WNV* Flavivirus* strains were quantified using the* Flavivirus* RT-qPCR with the WNV calibration curve and were subsequently identified by microarray analysis.

**Figure 3:**
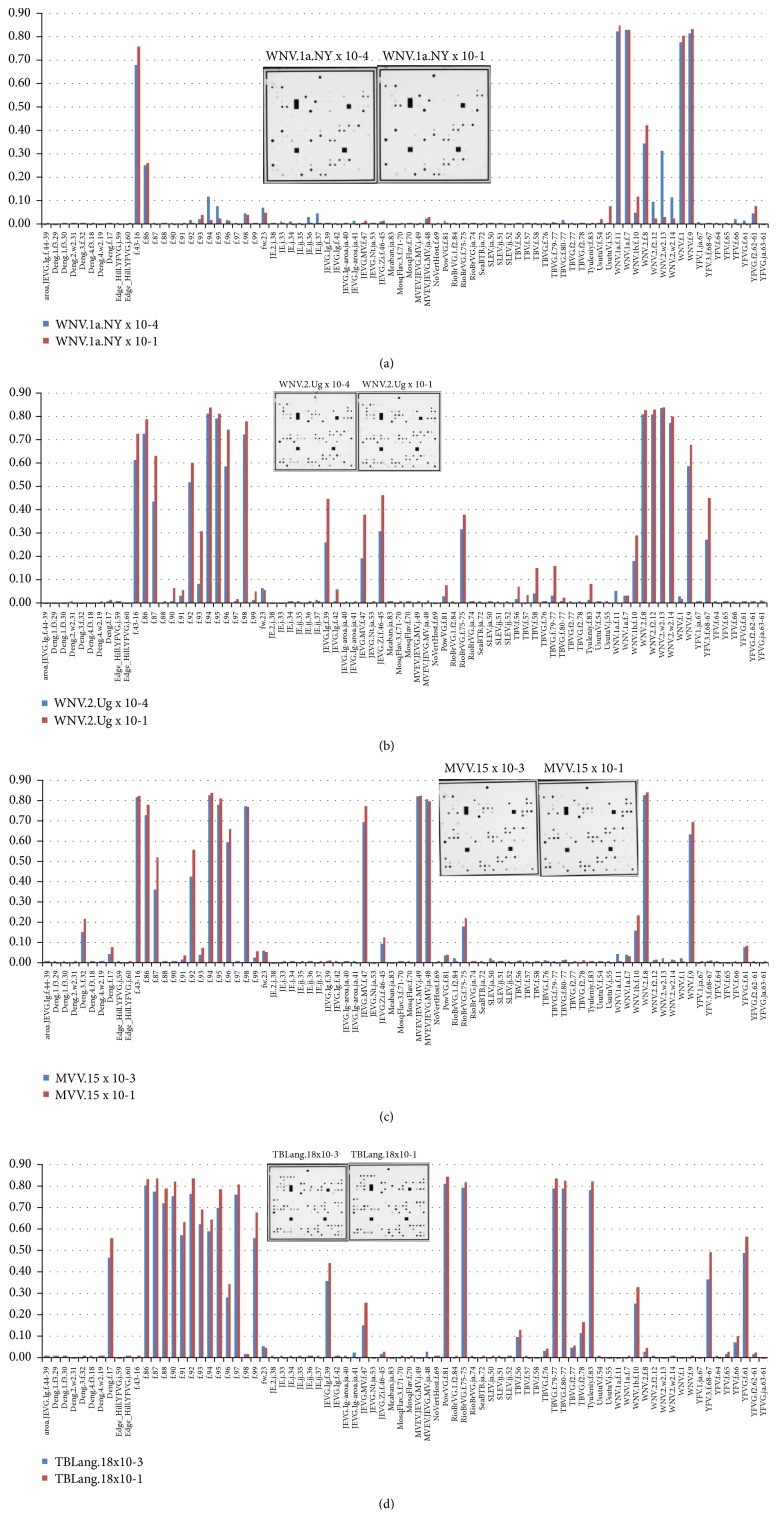
Hybridization patterns for different dilutions of viral samples. Cell culture supernatants: (a) West Nile virus lineage 1a, strain NY99 (WNV.1a.NY); red bars: sample diluted 10^−1^ and blue bars: diluted 10^−4^; (b) West Nile virus lineage 2, strain Uganda 1937 (WNV.2.Ug); red bars: 10^−1^ and blue bars: 10^−4^; (c) Murray Valley encephalitis virus (MVEV); red bars: 10^−1^ and blue bars: 10^−3^; (d) Tick-borne encephalitis virus, strain Langaat (TBLang); red bars: 10^−1^ and blue bars: 10^−3^. These data show that hybridization patterns are very sensitive to variation in the viral sequence, while remaining stable in a broad concentration range of viral RNA.

The combined RT-qPCR/microarray procedure was applied on the 26 reference virus strains and RT-qPCR positive field samples of diverse origin, in more than 300 hybridization experiments. Each analyzed reference virus produced a specific hybridization pattern that allowed discrimination. Most flaviviruses could be identified at the level of species, genotype, or even strain, following comparison of their hybridization patterns with those of reference samples. A compilation of four* Flavivirus* isolates each examined at two different RNA dilutions is shown in Figure 3. This figure reveals an important distinction of this microarray platform from well-known glass-slide arrays used for gene expression studies, meaning that the developed microarray is optimized to detect genetic (sequence) variations, rather than the concentration or relative quantity of amplicons. Thus, the present microarray signal intensity values are used solely for identification or classification, while quantification is performed in the preceding RT-qPCR step. As shown in Figure 3, the hybridization patterns are not significantly affected by quantitative variations in the viral load of the sample, but qualitative changes are readily visible when different strains of the same virus species are examined.

**Figure 4:**
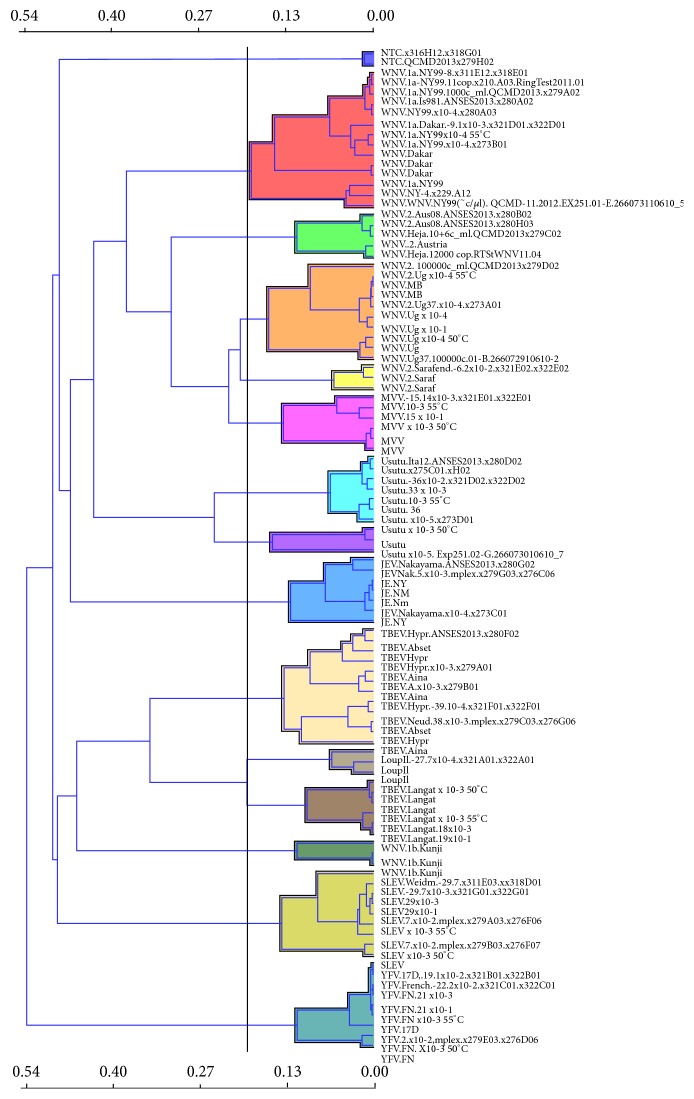
Cluster analysis of tested samples. Output of the tree widget in* Orange* software. A cluster analysis was conducted using the distances between the hybridization patterns of experimentally tested samples. This is the preferred method to visualize the identification of viruses in samples that contain only one* Flavivirus*.

**Figure 5:**
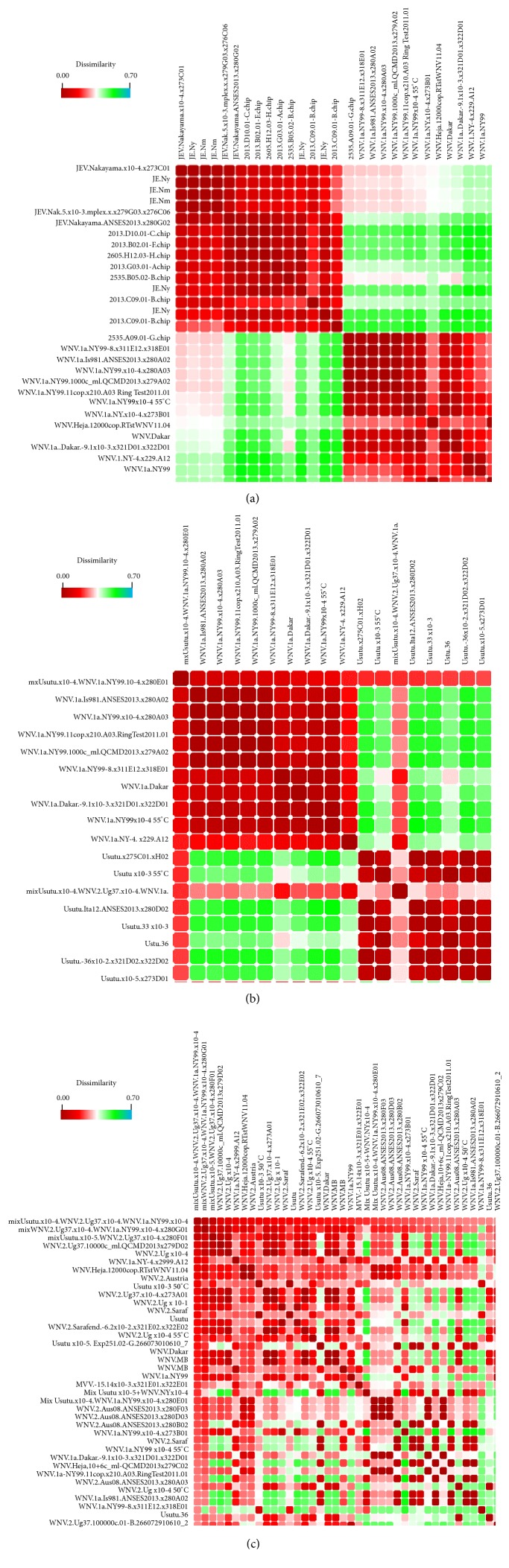
Presentation of experimental results processed by the* Orange's* heat-map-like widget showing an all-versus-all sample comparison. Calculated distances between intensity patterns of each pair of microarray results obtained from hybridization of sample amplicons are shown, with each cell representing the comparison of two microarray experiments. The dissimilarity color scale is shown at the top. Columns and rows are organized in the same order, making the diagonal an “identity” sample comparison. When one sample is selected by mouse-clicking, the map is immediately reorganized to show this sample in the upper row and left column followed by the most similar samples. (a) The selection of the “JEV.Nakayama.x10-4.x273C01” sample reveals a good separation of JEV samples from all other viral species. (b) The same heat map after selecting a sample containing a mix of diluted USUV and WNV1a RNA, which is easily recognized by a* framed* mosaic-like map. (c) A more complex mix, including USUV, WNV1, and WNV-2 RNA, was selected showing a more complex pattern that still permits the identification of the components (which can be additionally confirmed by the successive selection of each of the three standard WNV-1, WNV-2, and USUV to check that this mix clusters together with each of them).

In some cases, virus identification is already possible by visually comparing the signal patterns of the bar diagrams (e.g., those in Figure 3). However, given the complexity of the signals, it was necessary to include a computer-based solution for data processing.* Orange* was used to create a visual program (Figure S01) to import the raw data from the* Icono Clust* software, to define a set of experimental standards and identify viral samples by clustering (Figure 4). The procedure also permits visualization of mixed of* Flavivirus *infections in heat-map-like graphics (Figure 5). This capability represents another major advantage of the microarray compared to direct sequencing. Mixes of RNA from closely related* Flavivirus* strains were tested to explore the possibility of detection of coinfections in a given sample, or detection of the presence of more than one virus in tested pools. We have selected USUV and WNV-1a and WNV-2 (NY99 and Ug37) viruses to show that it is possible to unambiguously detect each component in a mix even of related viruses, such as lineages 1 and 2 of the WNV (Figures 5(b) and 5(c)).

The* Flavivirus* RT-qPCR screening was conducted on the 340 mosquito pools from Greece. PCR products of 13 mosquito pools (including all positive specimens, as well as six RT-qPCR-negative pools) underwent microarray analysis (Table 2), which revealed the presence of WNV lineage 2 sequences similar to the Austria strain in five of them (MPGr.01-MPGr.05, for ~1.5% of the total number of pools tested).* Culex* mosquitoes comprised all five WNV-positive pools. One pool yielded a strong positive result (Cq: 21.5), two were of mediums (Cq – 31.6 and 32.5), and two were of weak concentrations (Cq: 37.3 and 38.5). Sequencing of these five amplicons revealed identity to WNV isolates goshawk-Hungary/04 DQ116961, Nea Santa-Greece-2010, HQ537483 [42], and Italy 2013 KF647248, in four cases, while the fifth (the one with the highest Cq value) was not sequenceable.

The microarray also detected weak signals of insect-specific flaviviruses (ISFVs) in two of the pools tested. DNA sequencing revealed the presence of sequences with similarities to the isolate HU4528/07 of Marisma mosquito virus (JN603190, 93% nucleotide identity) in one* Aedes* sp. pool (MPGr.06). Additionally, a presumably new mosquito* Flavivirus* (with only 78% identity with GQ165809, the Nakiwogo virus strain from Uganda) was also detected in an* Anopheles *sp. pool (MPGr.07).* Flavivirus* RT-qPCR/microarray screening of the present panel was completed in less than three working days.

Additionally, other specimens were analyzed, including organs (such as brain, heart, liver, lung, kidney, and spleen) and blood from falcons [24], pheasants, blackbird, great grey owl, common kingfisher, and nearly 70 other avian species [6], as well as from mice, camels, horses, donkeys, cattle, humans, mosquitoes, and ticks. Only horse samples have caused artifacts in the RT-qPCR, showing low nonspecific signals (Cq > 33), thus effectively lowering the detection limit of RT-qPCR alone in horse samples to over 50 copies per reaction (data not shown).

## 4. Discussion

By designing the present combined RT-qPCR/microarray assay for detection, quantification, and identification of flaviviruses, a number of methodological problems have been solved. Using* VisualOligoDeg,* we selected primers and probes for the newly developed assay. The ability of a relatively accurate quantification during the RT-qPCR phase of the assay is one of its major advantages. Technically, the combined RT-qPCR/microarray assay is easy to handle, as only standard experience with real-time PCR and ELISA-like tests is required. We regularly achieved complete testing of samples in one working day, from RNA extraction to final visualization of the tree and interactive heat-map-like graphics.

The present assay permits classification and/or identification up to the (sub)lineage level, avoiding in most cases the need for sequencing. It has been shown to be as sensitive as species-specific RT-qPCRs and suitable for broad-range* Flavivirus *screening, as well as a confirmatory assay in both laboratory and field samples. The present study has also demonstrated that the assay can be efficiently used in arbovirus surveillance programs, for rapid screening and discrimination of flaviviruses, for example, in mosquito or animal specimens. In areas where numerous arbovirus strains of different virulence cocirculate, such as in Greece and other European countries, molecular identification of the circulating viruses is a necessity [10]. Especially for flaviviral zoonoses, phylogeography and identification of virulent strains are of utmost importance. The assay is also capable of detecting insect-specific flaviviruses. A report on the presence of ISFVs in* Culex* mosquitoes of Central Macedonia-Greece already exists [43]. Application of RT-qPCR/microarray testing revealed the presence of a virus strain with sequence similarities to Marisma mosquito virus, as well as a presumably novel mosquito* Flavivirus* sequence, in the same area of Greece. The detection of these ISFVs via the combined RT-qPCR/microarray protocol extends our knowledge on the presence of mosquito flaviviruses in Greece. The broad range of flaviviruses that are being tested simultaneously in this assay, in combination with its convenience and the minimum time required for obtaining the results, makes it a useful tool that potentially can be applied widely for surveillance and epidemiological surveys.

While our assay has been proven to be suitable for diagnostic and research laboratories, it represents an open system that can be further improved. In case of newly emerging pathogenic and genetically distinct strains, primer sequences could be further optimized using newly available genome sequences and/or improving probes of those species that were not in the focus of the present study (possibly DENV 4). The number of probes on the microarray is currently at 84 but could be increased to 500, using the present technology. Since we relied on the specifications of the commercial hybridization kit, we did not perform extensive optimization of the incubation times and temperature and will evaluate the possibility of using future versions of that kit [44]. A limitation of the present assay is the requirement of experimental hybridizations of known samples, which does not allow direct classification of unknowns. Nevertheless, the methodology is robust enough to allow the use of theoretical hybridization patterns, or those obtained in other laboratories, which could be a solution when a particular virus reference is not available. It should be emphasized that the tree constructed by the* Orange* tree widget using the distances between hybridization patterns is not a phylogenetic tree. The graphics just help to group samples according to sequence-based relatedness and thereby facilitates their identification. The* Orange *software also provides possibilities for classification using machine-learning methods, which have yet to be explored, but they have the potential to significantly improve the accuracy of the final report.

Compared to the diagnostic assays for* Flavivirus *detection published so far, our procedure is distinguished by its high degree of parallelity in detection of a wide range of virus species, strains, and their variations, which cannot be achieved through “one-dimensional” RT-PCR assays [21, 32, 45]. Previously published microarray or diagnostic chip approaches [20] lack the ease of operation of the ArrayStrip platform used here and cover only part of the range of viruses that we can identify [12, 14, 19, 46].

## 5. Conclusion

We have developed a combined RT-qPCR-microarray assay for high-throughput screening and identification of flaviviruses, including mixed infections of different species or strains. Our experience in analyzing field samples (from ticks and mosquito vectors and from human and animal samples of different sources) shows that the assay allows rapid and highly sensitive screening and identification of* Flavivirus* strains within one day. The assay has helped to overcome limitations in virological diagnosis due to lack of specificity or sensitivity in conventional and real-time RT-PCR protocols. Even if direct sequencing is used as genotyping tool, the developed microarray can be a used as a rapid complementary test to detect mixtures of different* Flavivirus* strains. The good performance of the assay was also confirmed, by correctly quantifying and identifying members of the* Flavivirus* genus in samples from international ring trials for quality assessment of nucleic acid amplification tests.

## Supplementary Material

Figure S01: The visual program PanFlavExpStdSampl. Schematic workflow—the visual program PanFlavExpStdSampl for the Orange software package. The scheme permits an interactive import of the experiments used as standards (known samples), which are being subsequently used to identify new or unknown samples. Figure S02: WNV titration. Figure S03: USUV titration. Table S01: Flavivirus-specific oligonucleotide probes, including spot number, position, probe name and probe sequence. Table S02: Quantification of flaviviruses in samples from the QCMD-2013 ring trial using the new Flavivirus RT-qPCR and a calibrated WNV NY99 RNA standard curve.

## Figures and Tables

**Table 1:** Thermal cycling profiles used in the *Flavivirus* RT-qPCR.

Standard (4.5 h, 25 *μ*L)	Fast (2.5 h, 25 *μ*L or 10 *μ*L)
(1) 50°C for 30 min (2) 95°C for 15 min (3) 95°C for 15 s (4) 55°C for 25 s + plate read (5) 72°C for 25 s + plate read (6) 80°C for 1 s + plate read (7) GOTO (3); 44 more times (8) 95°C for 1 min (9) Melting curve, 68 to 88°C, increment 0.1°C, 1 s + plate read (10) 4°C forever (optional) End	(1) 50°C for 30 min (2) 95°C for 15 min (3) 95°C for 15 s (4) 55°C for 20 s (5) 72°C for 20 s + plate read (6) GOTO (3); 44 more times (7) 95°C for 1 min (8) Melting curve, 68 to 86°C, increment 0.2°C, 1 s + plate read End

**Table 2:** Screening of mosquito pools collected in Greece. Review of the screening of the 340 mosquito pools (50 mosquitoes each) collected in Greece. All RT-qPCR positive and 6 RT-qPCR negative pools were tested further by microarray and direct sequencing. All other pools tested showed a Cq > 38 and Tm < 78°C.

Mosquito pool	Cq	Tm (°C)	Microarray	DNA sequencing result
MPGr.01	37.3	81.4	WNV.2 Austria	Nea Santa-Greece-2010 HQ537483
MPGr.02	31.6	81.6	WNV.2 Austria	Nea Santa-Greece-2010 HQ537483
MPGr.03	21.5	81.7	WNV.2 Austria	Nea Santa-Greece-2010 HQ537483
MPGr.04	32.5	81.9	WNV.2 Austria	Nea Santa-Greece-2010 (with 1 mutation)
MPGr.05	38.5	81.6	WNV.2 Austria	Negative
*MPGr.06*	*35.6*	*80,4*	*?*	*Marisma mosquito virus (93% identity)*
MPGr.07	39.6	81.0	Culex Flav	New Mosq Culex *Flavivirus* (78% identity)
MPGr.08	39.8	79.4	Negative	Negative
MPGr.09	41.4	83.0	Negative	Negative
MPGr.10	38.6	81.2	Negative	Negative (residual sequence: *Salmonella* sp.)
MPGr.11	40.1	82.4	Negative	Negative
MPGr.12	40.6	79.2	Negative	Negative
MPGr.13	39.6	80.7	Negative	Negative (residual sequence: *Pseudomonas* sp.)
