# Supplementary material for: A Novel Pan-Flavivirus Detection and Identification Assay Based on RT-qPCR and Microarray
Source: Biomed Res Int. 2017 May 24;2017:4248756. doi: 10.1155/2017/4248756 (PMC5463098; doi:10.1155/2017/4248756)
Supplement: Supplementary file 1 — Figure S01: The visual program PanFlavExpStdSampl. Schematic workflow—the visual program PanFlavExpStdSampl for the Orange software package. The scheme permits an interactive import of the experiments used as standards (known samples), which are being subsequently used to identify new or unknown samples. Figure S02: WNV titration. Figure S03: USUV titration. Table S01: Flavivirus-specific oligonucleotide probes, including spot number, position, probe name and probe sequence. Table S02: Quantification of flaviviruses in samples from the QCMD-2013 ring trial using the new Flavivirus RT-qPCR and a calibrated WNV NY99 RNA standard curve. [file 4248756.f1.pptx]

## Slide 1
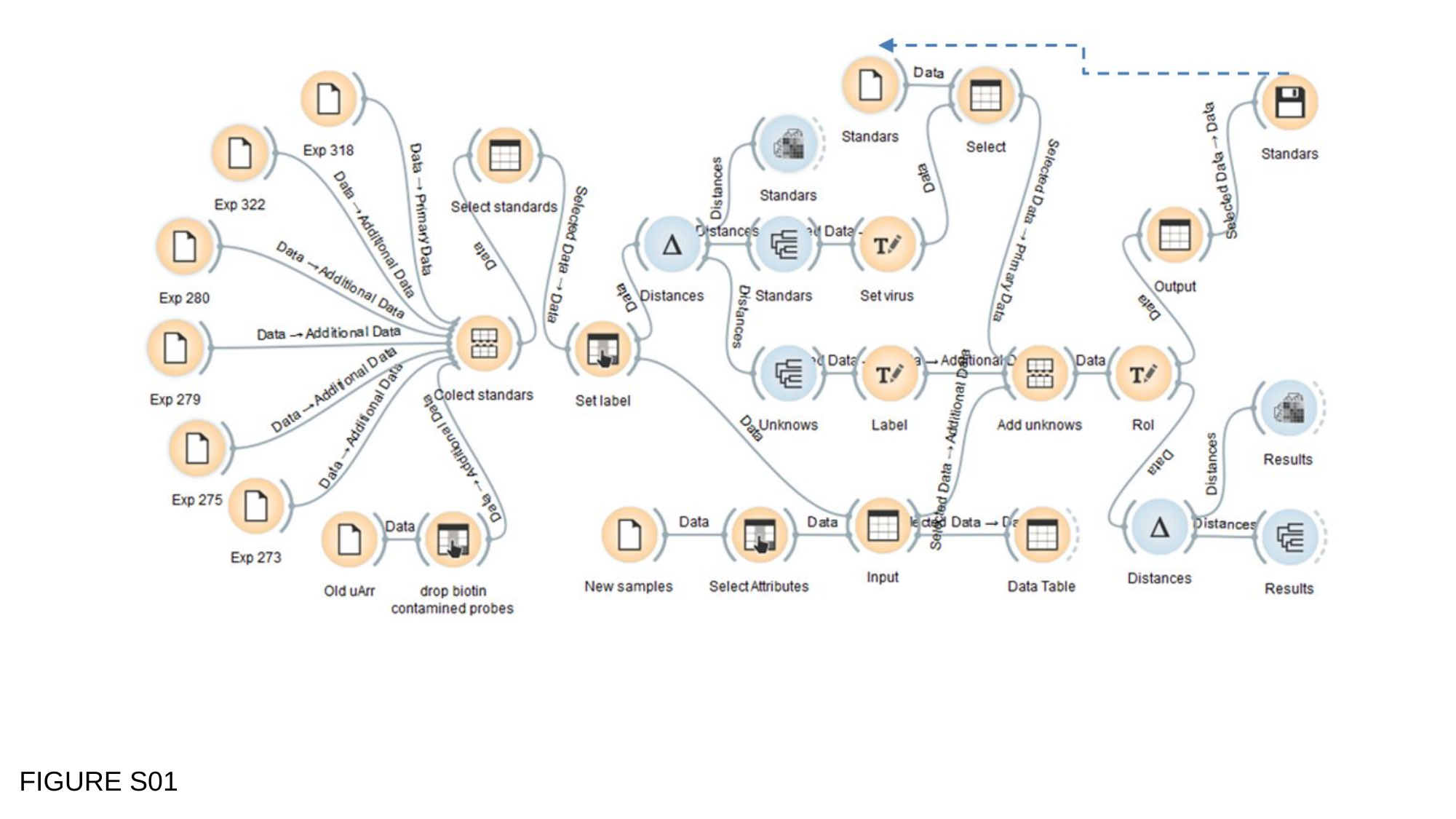

#
FIGURE S01

## Slide 2
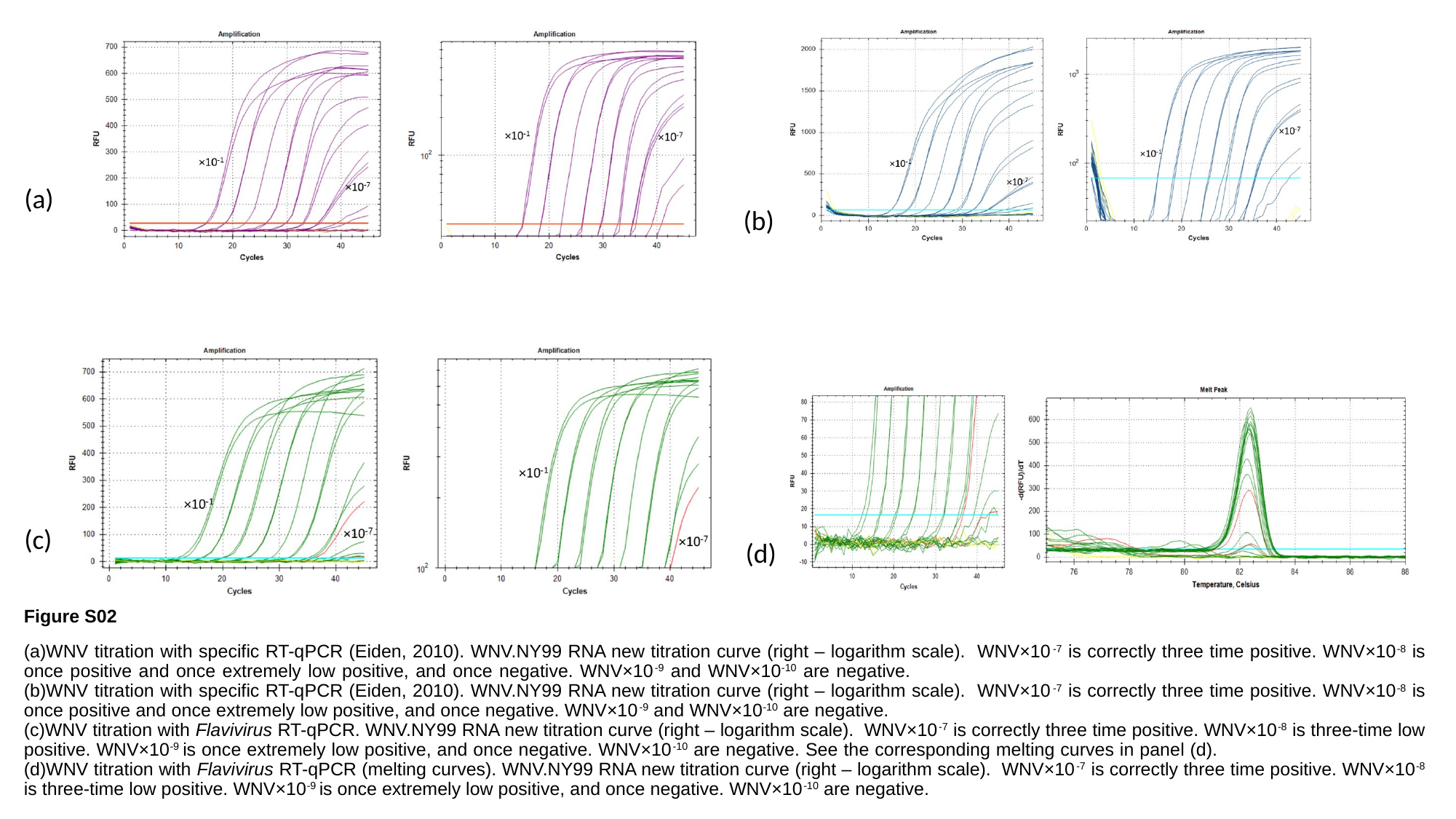

(a)
(b)
(c)
(d)
Figure S02
(a)WNV titration with specific RT-qPCR (Eiden, 2010). WNV.NY99 RNA new titration curve (right – logarithm scale). WNV×10-7 is correctly three time positive. WNV×10-8 is once positive and once extremely low positive, and once negative. WNV×10-9 and WNV×10-10 are negative.					 (b)WNV titration with specific RT-qPCR (Eiden, 2010). WNV.NY99 RNA new titration curve (right – logarithm scale). WNV×10-7 is correctly three time positive. WNV×10-8 is once positive and once extremely low positive, and once negative. WNV×10-9 and WNV×10-10 are negative.					 (c)WNV titration with Flavivirus RT-qPCR. WNV.NY99 RNA new titration curve (right – logarithm scale). WNV×10-7 is correctly three time positive. WNV×10-8 is three-time low positive. WNV×10-9 is once extremely low positive, and once negative. WNV×10-10 are negative. See the corresponding melting curves in panel (d). (d)WNV titration with Flavivirus RT-qPCR (melting curves). WNV.NY99 RNA new titration curve (right – logarithm scale). WNV×10-7 is correctly three time positive. WNV×10-8 is three-time low positive. WNV×10-9 is once extremely low positive, and once negative. WNV×10-10 are negative.

## Slide 3
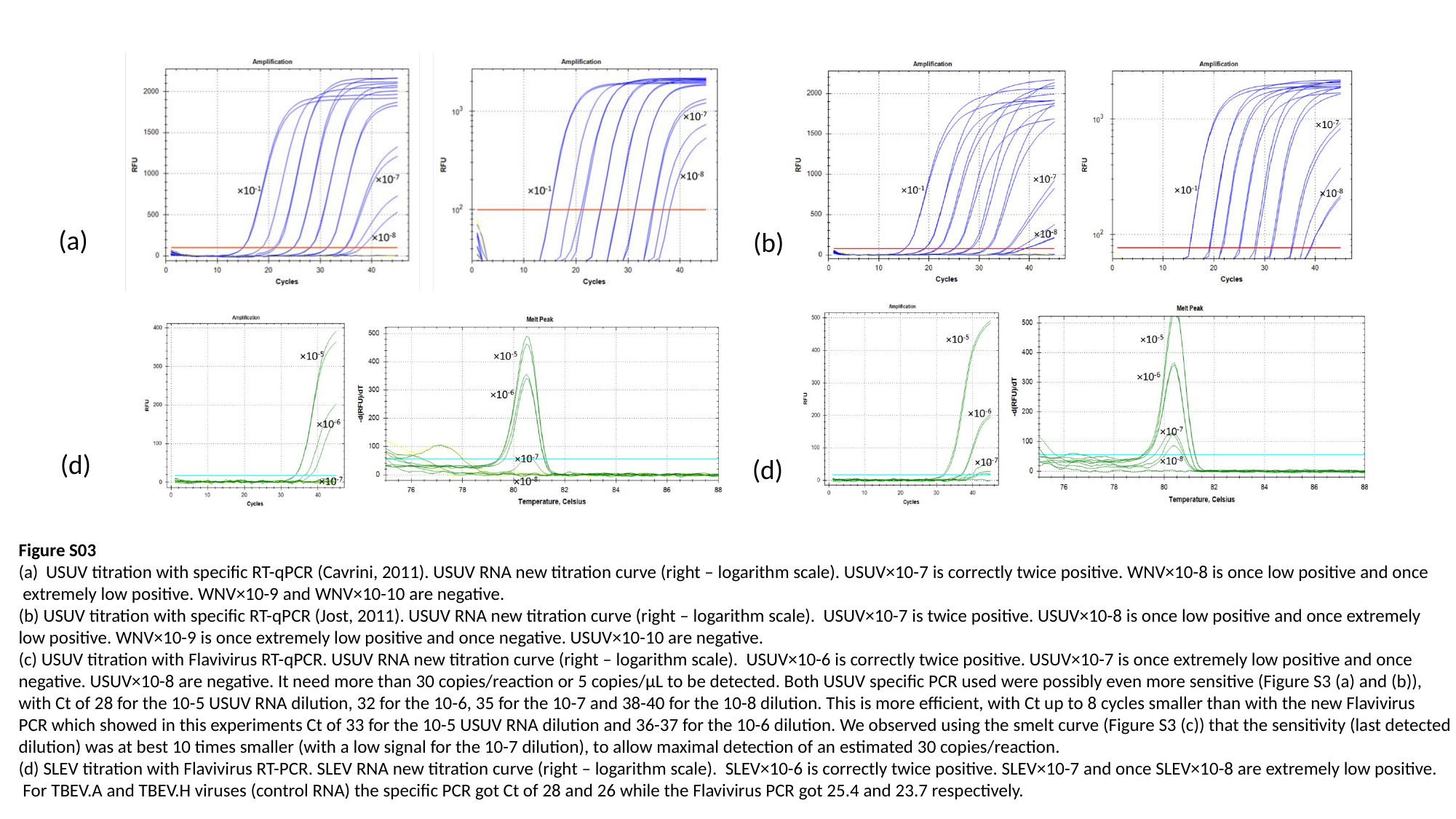

(a)
(b)
(d)
(d)
Figure S03
USUV titration with specific RT-qPCR (Cavrini, 2011). USUV RNA new titration curve (right – logarithm scale). USUV×10-7 is correctly twice positive. WNV×10-8 is once low positive and once
 extremely low positive. WNV×10-9 and WNV×10-10 are negative.
(b) USUV titration with specific RT-qPCR (Jost, 2011). USUV RNA new titration curve (right – logarithm scale). USUV×10-7 is twice positive. USUV×10-8 is once low positive and once extremely
low positive. WNV×10-9 is once extremely low positive and once negative. USUV×10-10 are negative.
(c) USUV titration with Flavivirus RT-qPCR. USUV RNA new titration curve (right – logarithm scale). USUV×10-6 is correctly twice positive. USUV×10-7 is once extremely low positive and once
negative. USUV×10-8 are negative. It need more than 30 copies/reaction or 5 copies/µL to be detected. Both USUV specific PCR used were possibly even more sensitive (Figure S3 (a) and (b)),
with Ct of 28 for the 10-5 USUV RNA dilution, 32 for the 10-6, 35 for the 10-7 and 38-40 for the 10-8 dilution. This is more efficient, with Ct up to 8 cycles smaller than with the new Flavivirus
PCR which showed in this experiments Ct of 33 for the 10-5 USUV RNA dilution and 36-37 for the 10-6 dilution. We observed using the smelt curve (Figure S3 (c)) that the sensitivity (last detected
dilution) was at best 10 times smaller (with a low signal for the 10-7 dilution), to allow maximal detection of an estimated 30 copies/reaction.
(d) SLEV titration with Flavivirus RT-PCR. SLEV RNA new titration curve (right – logarithm scale). SLEV×10-6 is correctly twice positive. SLEV×10-7 and once SLEV×10-8 are extremely low positive.
 For TBEV.A and TBEV.H viruses (control RNA) the specific PCR got Ct of 28 and 26 while the Flavivirus PCR got 25.4 and 23.7 respectively.

## Slide 4
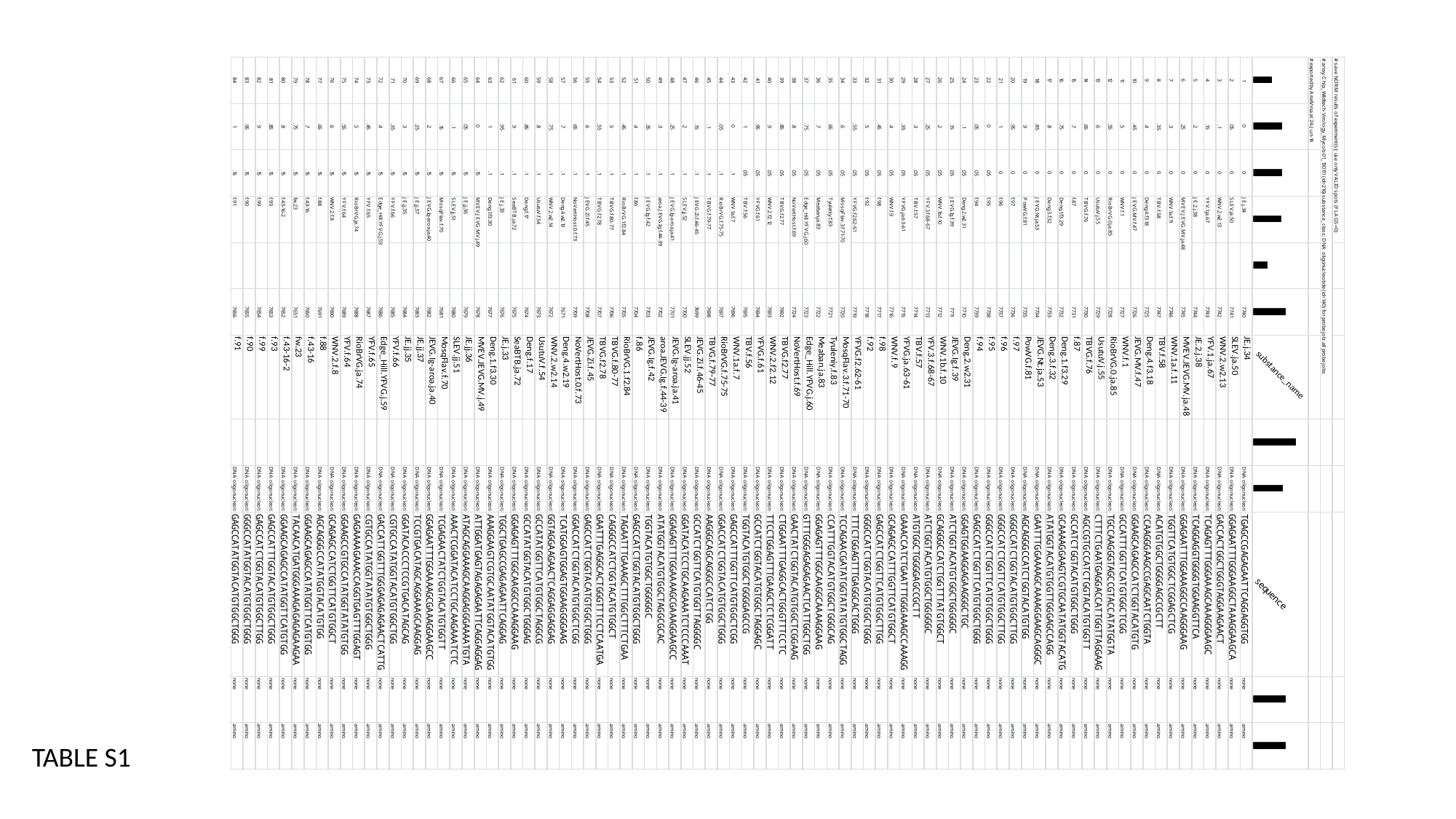

TABLE S1

## Slide 5
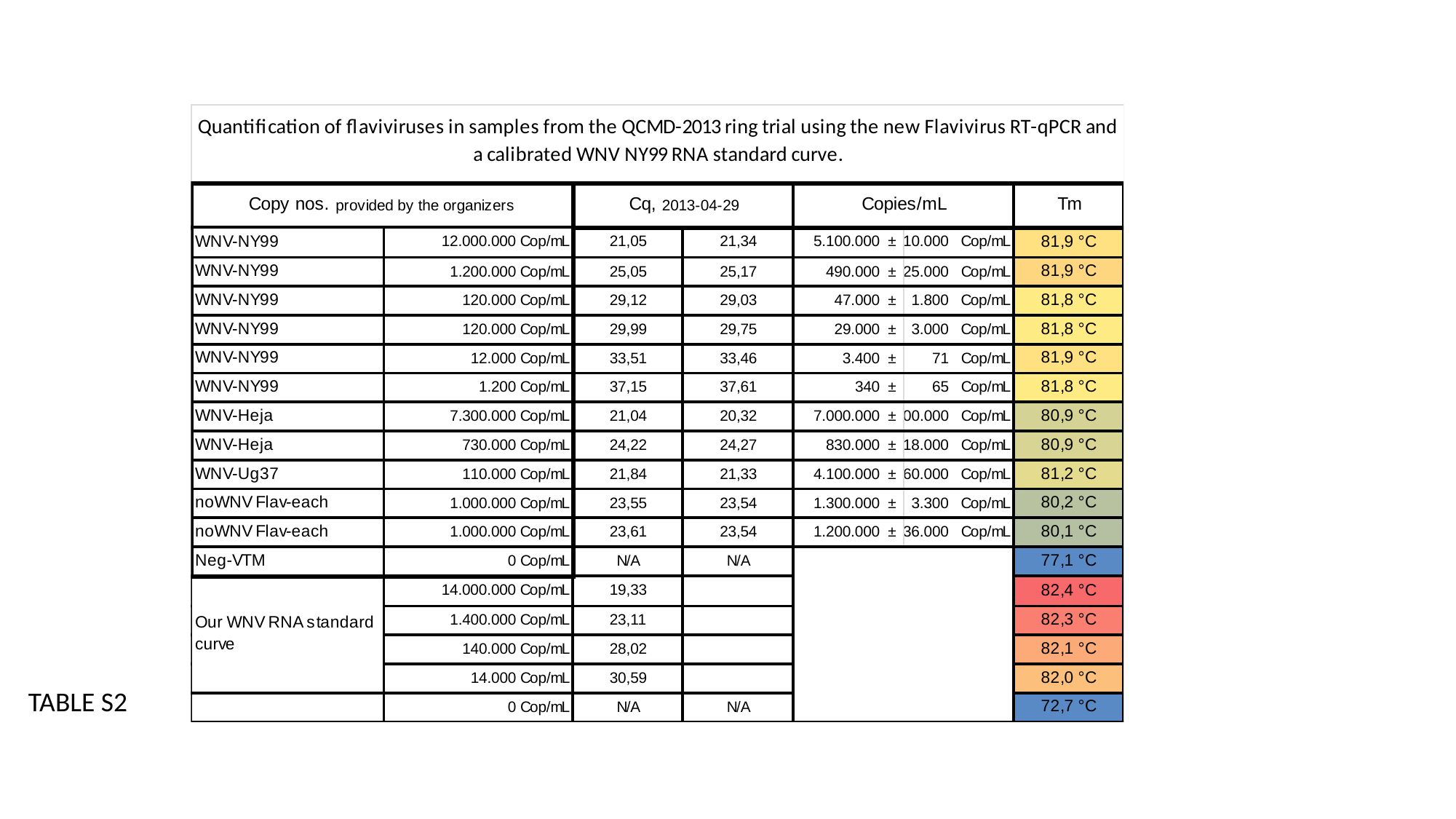

TABLE S2
